# Emerging Threat of *Acinetobacter radioresistens* Infection in Immunocompromised Patients

**DOI:** 10.1155/crdi/2388640

**Published:** 2026-02-20

**Authors:** Jyotsna Mary George, Guiseppe Calandrino, Rushdah Malik

**Affiliations:** ^1^ Henry Ford Providence Southfield Campus, Southfield, Michigan, USA

**Keywords:** *Acinetobacter radioresistens*, antimicrobial resistance, bacteremia, MALDI-TOF, VERIGENE

## Abstract

*Acinetobacter radioresistens* is an uncommon human pathogen rarely reported to cause bacteremia. Its accurate identification is crucial yet challenging, with implications for antimicrobial stewardship due to its potential to harbor carbapenem resistance genes. We present the case of a 77‐year‐old male with metastatic lung adenocarcinoma, COPD, and dementia who presented with acute hypoxic respiratory failure and septic shock. Initial empiric antibiotics were vancomycin and cefepime. Gram stain of positive blood cultures revealed Gram‐negative coccobacilli. The VERIGENE BC‐GN microarray system identified an *Acinetobacter* species, negative for common resistance markers, which was subsequently confirmed as pan‐susceptible *Acinetobacter radioresistens* by MALDI‐TOF mass spectrometry. The patient’s antibiotic regimen was de‐escalated to intravenous ampicillin‐sulbactam, to which he initially responded. His course was later complicated by recurrent respiratory failure, and his family transitioned him to comfort care. He expired shortly after withdrawal of life support. This case underscores the emerging clinical significance of *A. radioresistens* as an opportunistic pathogen in immunocompromised hosts. It highlights the utility of rapid molecular diagnostics for precise pathogen identification and antimicrobial stewardship, while also illustrating the critical role of confirmatory methods like MALDI‐TOF. Vigilance for less common *Acinetobacter* species is warranted in patients with significant comorbidities.

## 1. Introduction

The genus *Acinetobacter* consists of Gram‐negative coccobacilli that are a growing concern as a cause of hospital‐acquired infections. While *Acinetobacter baumannii* is the most prevalent and notorious species due to its multidrug resistance, other species within the genus are increasingly recognized as opportunistic pathogens. *Acinetobacter radioresistens* is one such organism, typically considered to have low virulence but capable of causing serious infections in immunocompromised hosts. Accurate and rapid identification of bacterial pathogens in bloodstream infections is crucial for guiding appropriate antibiotic therapy. This case report describes a case of bacteremia and pneumonia caused by *A. radioresistens* in a patient with multiple comorbidities, highlighting the diagnostic pathway and the importance of advanced microbiological techniques in clinical management. This case report has been prepared in accordance with the CARE guidelines (see Supporting Information [available here]).

## 2. Presentation of Case

**Figure 1:**
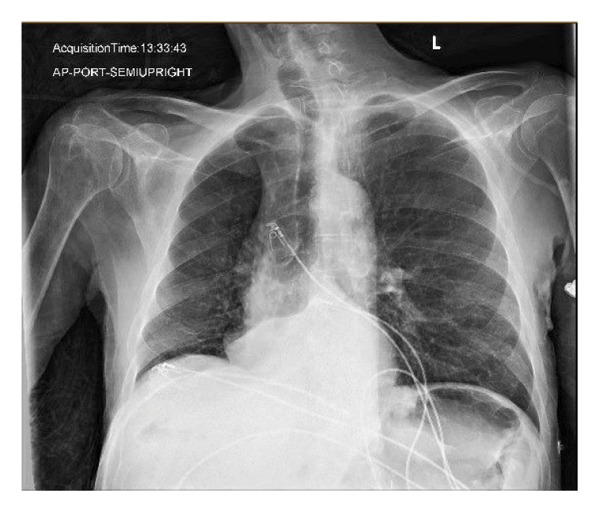
Retrocardiac opacity consistent with lobar atelectasis, possibly postobstructive.

**Figure 2:**
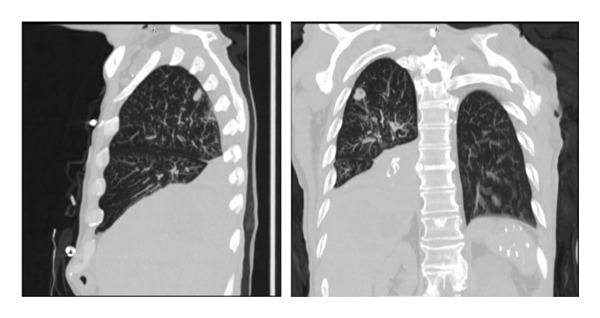
Complete opacification of the right lower lobe of the lung, and a 1.5 cm solid pulmonary nodule within the right upper lobe of the lung with multiple smaller nodules throughout the right lung.

A 77‐year‐old male with adenocarcinoma of the lung and liver metastasis, presented from a group home due to shortness of breath. His cancer diagnosis was recent, and he had not yet undergone chemotherapy or radiation. His additional comorbidities included COPD, dementia, and history of alcohol abuse. Physical examination revealed he was afebrile, tachypneic (31 breaths/min), hypoxic (85% saturation on room air), and hypotensive (77/61 mm Hg). Crackles were noted in the upper right lung lobe. He was initially placed on a nonrebreather mask, but his respiratory support was escalated to BIPAP therapy. Due to progressive fatigue on BIPAP, he was subsequently intubated. His laboratory evaluation was significant for leukocytosis of 14.89 × 10^3^/μL, hypoglycemia 55 mg/dL, acute kidney injury (creatinine 2.5 mg/dL; baseline 0.8 mg/dL), hypernatremia 162 mg/dL, hyperkalemia 5.5 mg/dL, anion gap metabolic acidosis (AGAP 30, bicarb 10 mmol/L, lactic acid 18.5 mmol/L), and transaminitis (Alk Phos 440 U/L, AST 552 U/L, ALT 117 U/L). Venous blood gas showed metabolic acidosis with a pH of 7.15, pCO_2_ 32 mmHg, bicarb 11.1 mmol/L, and lactic acid of 17.0 mmol/L. Chest X‐ray (Figure 1) revealed a retrocardiac opacity consistent with lobar atelectasis, potentially postobstructive. Noncontrast CT of the thorax (Figure 2) revealed complete opacification of the right lower lobe and a 1.5 cm solid pulmonary nodule in the right upper lobe, along with multiple smaller nodules throughout the right lung. Noncontrast CT of the abdomen and pelvis showed findings of colitis and multiple hypoattenuating liver lesions, suggestive of cholangiocarcinoma or metastasis. The patient was admitted to the medical intensive care unit for acute hypoxic respiratory failure requiring mechanical ventilation. Vasopressors were initiated for persistent hypotension despite fluid resuscitation. Empiric antibiotic therapy with vancomycin (1000 mg IVPB) and cefepime (1000 mg IVPB) was initiated.

**Figure 3:**
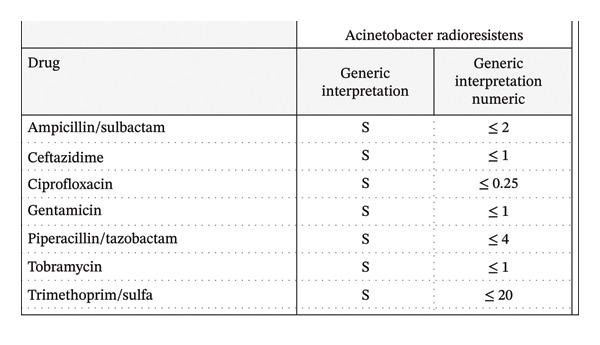
Antimicrobial susceptibility profile of the *Acinetobacter radioresistens* isolate from the patient’s blood culture, as determined by minimum inhibitory concentrations (MICs) according to CLSI guidelines.

Gram stain of one of two positive blood cultures (aerobic bottle) obtained on admission was positive but initially untypable by standard microbial testing. Tracheal aspirate and sputum cultures grew *Klebsiella pneumoniae.* VERIGENE molecular testing of the blood cultures identified an *Acinetobacter* species [1] that was negative for resistance markers CTX‐M, KPC, IMP, NDM, OXA, and VIM. This was subsequently identified as *Acinetobacter radioresistens* by MALDI‐TOF mass spectrometry, demonstrating pan‐susceptibility to ampicillin/sulbactam, ceftazidime, ciprofloxacin, gentamicin, piperacillin/tazobactam, tobramycin, and trimethoprim/sulfamethoxazole. Antimicrobial susceptibility testing was performed by the VITEK 2 system according to the Clinical and Laboratory Standards Institute (CLSI) guidelines (Figure 3). He was treated with an eight‐day course of intravenous ampicillin‐sulbactam (3 g every 8 h) and a two‐day course of metronidazole (500 mg every 8 h).

The patient initially showed improvement following antibiotic initiation and was weaned off vasopressor and oxygen support. His subsequent clinical course was complicated by a sudden hypoxemic respiratory failure, again necessitating vasopressor support and mechanical ventilation. Given the patient’s poor prognosis, diminished quality of life, and multiple comorbidities, his family elected for hospice and comfort care and to withdraw life support. The patient expired shortly after the withdrawal of life support. It remained unclear whether the patient’s death was attributable to the infectious process, given his initial improvement with antibiotics, or to the progression and complications of his metastatic disease. A detailed timeline of the patient’s clinical course is provided in Table 1.

**TABLE 1:** Timeline of clinical course and interventions.

Hospital day	Clinical event	Diagnostic findings	Interventions and management
Day 1	Presentation from group home with shortness of breath, hypotension, and hypoxia	Labs: Leukocytosis, AKI, lactic acidosis. Imaging: CXR/CT with RLL opacification	Admission to MICU, intubation, vasopressors initiated. Empiric antibiotics: Vancomycin and Cefepime. Blood cultures drawn
Day 2	Blood cultures are reported as positive	VERIGENE BC‐GN: *Acinetobacter* species identified (no resistance markers detected)	Continued empiric antibiotics and supportive care
Day 4	Initial clinical improvement noted	MALDI‐TOF and VITEK 2: Confirmed as pan‐susceptible *A. radioresistens*	Antibiotic regimen de‐escalated to IV ampicillin/sulbactam
Day 5–11	Patient improved, weaned off vasopressors and oxygen support. Successfully extubated		Completed 8‐day course of targeted IV ampicillin/sulbactam
Day 12	Sudden clinical deterioration with recurrent hypoxemic respiratory failure		Re‐intubation and vasopressor support resumed. Family discussion regarding poor prognosis. Transition to comfort care. Withdrawal of life support. Patient expires

## 3. Discussion

*Acinetobacter* are aerobic, oxidase‐negative, Gram‐negative, nonmotile coccobacilli. While generally considered to have low virulence, they are capable of causing significant infections in immunocompromised and neutropenic patients [2]. Risk factors for *Acinetobacter* infections include prolonged ICU stays, prolonged antibiotic exposure, mechanical ventilation, central venous catheter use, and hemodialysis [3]. *Acinetobacter* infections are associated with high morbidity and mortality rates, reportedly up to 70% in patients with multiorgan dysfunction [3]. To our knowledge, this represents the tenth reported case of *Acinetobacter radioresistens* isolated from culture, with seven of these cases reportedly achieving successful treatment (Table 2).

**TABLE 2:** Reported cases of *Acinetobacter radioresistens* isolated from clinical cultures, including the present case, with treatment outcomes demonstrating successful management in seven of ten documented cases.

Case	Age	Sex	Comorbidities	Treatment	Outcome
Wang et al. [2]	71	Female	Adenocarcinoma of the lung	Ampicillin‐sulbactam 3 g q6h for 14 days	Deceased
Lopes et al. [4]	71	Male	Alzheimer’s, Parkinson’s	Ceftriaxone	Survived
Visca et al. [5]	32	Female	HIV	Ciprofloxacin 400 mg BID for 14 days	Survived
Tan et al. [6]	55	Male	Tobacco abuse	IV ampicillin‐sulbactam 3 g q6h for 1 week followed by PO ampicillin‐sulbactam 750 mg BID for 1 week	Survived
Brady et al. [7]	53	Female	Li‐Fraumeni syndrome, colon/breast/adrenal/bone cancer	Ceftriaxone and ampicillin‐sulbactam	Survived
	60	Male	Diabetes, ESRD	Cefepime	Survived
Savov et al. [8]	85	Male	COPD, CHF	Ceftriaxone 1 g BID and Gentamicin 80 mg	Deceased
Verma et al. [9]	61	Male	COPD, Hepatitis C	Ampicillin‐sulbactam for 14 days	Survived
Lazarev et al. [10]	83	Male	CHF, CAD, COPD	Ceftriaxone 2 g and azithromycin 500 mg	Survived
Our case	77	Male	Adenocarcinoma of the lung with metastasis to the liver	Ampicillin‐sulbactam 3 g q6h for 8 days and Flagyl 500 mg q8h for 2 days	Deceased

For timely and appropriate antibiotic administration, accurate and rapid identification of bacterial pathogens in bloodstream infections is crucial. In this case, *Acinetobacter radioresistens* was identified using the VERIGENE System [11]^,^ which is a multiplexed nucleic acid microarray platform that allows for the simultaneous detection of different pathogens and resistance markers directly from positive blood cultures. This provides results faster than traditional culture‐based methods and consists of automated nucleic acid extraction, hybridization to species‐specific probes on a microarray, and nanoparticle‐based detection.

The ability of the VERIGENE system for detecting *Acinetobacter radioresistens* at the species level reliably depends on the specific panel used. The VERIGENE Gram‐Negative Blood Culture (BC‐GN) panel usually targets common bloodstream pathogens. The inclusion and validation of probes that are specific for *A. radioresistens* can vary. The successful identification in this case supports the evidence that the panel utilized has the capability to detect this species. However, if clinical suspicion for less common organisms arises, confirmation with alternative methods may be warranted.

The most suitable methods for accurate identification of *Acinetobacter radioresistens* currently include conventional culture with biochemical identification [12], which is the foundational approach, and Matrix‐Assisted Laser Desorption/Ionization Time‐of‐Flight Mass Spectrometry (MALDI‐TOF MS) [13], which is known for its rapid and generally accurate identification of *Acinetobacter* species.

High resolution for species identification can be obtained through molecular methods such as PCR and sequencing of housekeeping genes [14] (e.g., *rpoB*, *gyrB*, and 16S rRNA). Even though the VERIGENE system serves as a rapid diagnostic tool, it is important to be aware of the coverage of the specific assay used for common pathogens and the need for supplementary methods for instances where unexpected isolates like *A. radioresistens* are encountered.

*Acinetobacter baumannii* has become a significant cause of hospital‐associated infections in recent years [15]. The *Acinetobacter* genus exhibits intrinsic resistance to ampicillin, first‐ and second‐generation cephalosporins, aztreonam, and ertapenem. The acquisition and expression of carbapenem‐hydrolyzing oxacillinases contributes to the carbapenem resistance in *Acinetobacter baumannii*. *Acinetobacter radioresistens* isolates have the *bla*‐OXA‐23 gene chromosomally encoded, which is implicated in the carbapenem resistance [16, 17].

*Acinetobacter radioresistens* is rarely identified as the causative agent of bacterial pneumonia. They are commonly found in the environment and are known to colonize human skin and the respiratory tract. While acknowledging the presence of *Klebsiella pneumoniae* in the patient’s pulmonary samples, which could contribute to the pneumonia, its isolation from the bloodstream makes *Acinetobacter radioresistens* is a clinically significant finding. Even though it has low virulence, its presence in an immunocompromised patient with severe pneumonia and sepsis, coupled with a positive blood culture confirmed by molecular methods and an initial rapid response to targeted antibiotics (ampicillin‐sulbactam, to which the *A. radioresistens* was fully susceptible), suggests it was the likely cause of bacteremia and sepsis in this setting. The critical clinical presentation of the patient aligns with a significant bloodstream infection, making simple blood culture contamination less likely in this context. The ability of this species to harbor the carbapenem resistance genes throws light on a significant pathway through which less virulent *Acinetobacter* species can contribute to the escalating antimicrobial resistance problem. Even though only one blood culture was positive for *Acinetobacter radioresistens,* the severity of the clinical presentation combined with the molecular identification of the species and the initial improvement to antibiotics, supports the diagnosis of *A. radioresistens* bacteremia.

In the case presented, the patient had several risk factors for *Acinetobacter* infection, including immunocompromised status, metastatic disease, mechanical ventilation, and ICU admission. Having any one of these risk factors increases the probability of acquiring an *Acinetobacter* infection. Comparison with other reported cases of *Acinetobacter radioresistens* isolation reveals that most, if not all, patients had comorbidities predisposing them to pulmonary compromise. This includes lung adenocarcinoma, COPD, Alzheimer’s/Parkinson’s (associated with increased risk of aspiration), tobacco use and immunocompromised status. Despite being rare, clinicians should remain vigilant for *Acinetobacter radioresistens* infections in patients with the risk factors mentioned and to tailor antibiotic therapy accordingly to reduce disease burden. Given that these infections mostly result from colonization and nosocomial spread [3], there should be measures taken to prevent colonization through appropriate isolation measures. In the absence of these measures, it can contribute to the further emergence of carbapenem‐resistant *Acinetobacter* species.

## 4. Conclusion

Our case highlights *Acinetobacter radioresistens* as a cause of bacteremia and significant pneumonia necessitating mechanical ventilation. Accurate identification was achieved using VERIGENE, a nucleic acid microarray capable of rigorously detecting pathogens and resistance markers. We highlight the importance of precise identification of pathogens, including profiling antibiotic resistance, in the management of critically ill patients. Molecular assays, such as VERIGENE, should be incorporated as an integral step when performing a comprehensive evaluation of such patients. This will allow us to understand the pathogenesis of *Acinetobacter radioresistens*, which may become more prevalent due to the increasing incidence of poor antimicrobial stewardship. We would also like to highlight the presence of *Acinetobacter radioresistens* infections in those with comorbidities which affect the lung. Further investigation is needed to determine if these comorbidities independently increase the risk of acquiring *Acinetobacter radioresistens* infection, or if their increased susceptibility is associated with their need for higher levels of care such as mechanical ventilation and ICU care.

## Funding

This research received no specific grant from any funding agency in the public, commercial, or not‐for‐profit sectors.

## Ethics Statement

As the data was fully de‐identified and the report was retrospective in nature, patient consent was not obtained. Patient privacy and confidentiality were maintained throughout the preparation of this case report.

## Consent

No written consent has been obtained from the patients as there is no patient identifiable data included in this case report.

## Conflicts of Interest

The authors declare no conflicts of interest.

## Supporting Information

The CARE checklist for this case report is provided as a supporting file (CARE Checklist.pdf).

## Supporting information


**Supporting Information** Additional supporting information can be found online in the Supporting Information section.

## Data Availability

The data supporting the findings of this case report are available within the article. Further details can be shared upon reasonable request from the corresponding author while protecting patient confidentiality.

## References

[1] Fournier P. E. , Richet H. , and Weinstein R. A. , The Epidemiology and Control of *Acinetobacter baumannii* in Health Care Facilities, Clinical Infectious Diseases. (2006) 42, no. 5, 692–699, 10.1086/500202, 2-s2.0-33144460009. 16447117

[2] Wang T. , Costa V. , Jenkins S. G. , Hartman B. J. , and Westblade L. F. , *Acinetobacter radioresistens* Infection With Bacteremia and Pneumonia, IDCases. (2019) 15, 10.1016/j.idcr.2019.e00495, 2-s2.0-85062395261. PMC6411504 30906692

[3] Playford E. G. , Craig J. C. , and Iredell J. R. , Carbapenem-Resistant *Acinetobacter baumannii* in Intensive Care Unit Patients: Risk Factors for Acquisition, Infection and Their Consequences, Journal of Hospital Infection. (2007) 65, no. 3, 204–211, 10.1016/j.jhin.2006.11.016. 17254667

[4] Lopes M. C. , Évora B. S. , Cidral T. A. et al., Bloodstream Infection by Acinetobacter Radioresistens: the First Case Report in Brazil, Jornal Brasileiro de Patologia e Medicina Laboratorial. (2019) 55, 667–672, https://www.scielo.br/j/jbpml/a/MVHWHLmMCHJkbxgd3nrKMVK/?format=html%26lang=en.

[5] Visca P. , Petrucca A. , De Mori P. et al., Community-Acquired *Acinetobacter radioresistens* Bacteremia in an HIV-Positive Patient, Emerging Infectious Diseases. (2001) 7, no. 6, 1032–1035, https://www.ncbi.nlm.nih.gov/pmc/articles/PMC2631918/pdf/11747736.pdf, 10.3201/eid0706.010621, 2-s2.0-0035202733. 11747736 PMC2631918

[6] Tan T. , Yh Y. H. , Quah Q. et al., Community-Acquired Pneumonia With *Acinetobacter radioresistens* Bacteremia in an Immunocompetent Host: A Case Report, Asian Pacific Journal of Tropical Medicine. (2019) 12, no. 6, 10.4103/1995-7645.261308, 2-s2.0-85068708556.

[7] Brady A. C. , Lewis J. S. , and Pfeiffer C. D. , Rapid Detection of *bla*OXA in Carbapenem-Susceptible *Acinetobacter radioresistens* Bacteremia Leading to Unnecessary Antimicrobial Administration, Diagnostic Microbiology and Infectious Disease. (2016) 85, no. 4, 488–489, 10.1016/j.diagmicrobio.2016.04.025, 2-s2.0-84970044578. 27236714

[8] Savov E. , Pfeifer Y. , Wilharm G. et al., Isolation of *Acinetobacter radioresistens* From a Clinical Sample in Bulgaria, The Journal of Global Antimicrobial Resistance. (2016) 4, 57–59, 10.1016/j.jgar.2015.10.008, 2-s2.0-84949818828. 27436395

[9] Verma R. R. and Baroco B. , *Acinetobacter radioresistens* Septicemia Associated With Pneumonia in a Patient With Chronic Obstructive Pulmonary Disease and Hepatitis C, Infectious Diseases in Clinical Practice. (2017) 25, no. 4, e00159–e13, 10.1097/IPC.0000000000000159, 2-s2.0-84920874079.

[10] Lazarev A. , Hyun J. , Sanchez J. L. , and Verda L. , Community-Acquired *Acinetobacter radioresistens* Bacteremia in an Immunocompetent Host, Cureus. (2022) 14, no. 9, 10.7759/cureus.29650. PMC9612910 36321031

[11] Jung J. , Kim S. H. , Lee S. et al., Rapid Identification of Gram-negative Bacteria and Their Resistance Genes From Positive Blood Cultures Using the VERIGENE BC-GN Assay, Diagnostic Microbiology and Infectious Disease. (2014) 79, no. 4, 434–438.

[12] Koneman E. W. , Allen S. D. , Janda W. M. , Schreckenberger P. C. , and Winn W. C. , Koneman’s Color Atlas and Textbook of Diagnostic Microbiology, 2006, 6th edition, Lippincott Williams & Wilkins.

[13] Croxatto A. , Prod′hom G. , and Greub G. , Applications of MALDI-TOF Mass Spectrometry in Clinical Microbiology, FEMS Microbiology Reviews. (2012) 36, no. 2, 380–407. 22092265 10.1111/j.1574-6976.2011.00298.x

[14] Clarridge J. E. , Impact of Molecular Methods on Clinical Microbiology, Clinical Microbiology Reviews. (2004) 17, no. 4, 829–851.

[15] Brady M. F. , Jamal Z. , and Pervin N. , Acinetobacter, StatPearls [Internet], 2023, StatPearls Publishing, Treasure Island (FL), https://www.ncbi.nlm.nih.gov/books/NBK430784/. 28613535

[16] Garnacho-Montero J. and Amaya-Villar R. , Multiresistant *Acinetobacter baumannii* Infections: Epidemiology and Management, Current Opinion in Infectious Diseases. (2010) 23, no. 4, 332–339, 10.1097/QCO.0b013e32833ae38b, 2-s2.0-77954888053. 20581674

[17] Poirel L. , Figueiredo S. , Cattoir V. , Carattoli A. , and Nordmann P. , *Acinetobacter radioresistens* as a Silent Source of Carbapenem Resistance for *Acinetobacter* spp, Antimicrobial Agents and Chemotherapy. (2008) 52, no. 4, 1252–1256, 10.1128/AAC.01304-07, 2-s2.0-42049101777. 18195058 PMC2292503

