# Supplementary material for: Emerging Threat of Acinetobacter radioresistens Infection in Immunocompromised Patients
Source: Case Rep Infect Dis. 2026 Feb 20;2026:2388640. doi: 10.1155/crdi/2388640 (PMC12921414; doi:10.1155/crdi/2388640)
Supplement: Supplementary file 1 — Supporting Information Additional supporting information can be found online in the Supporting Information section. [file CRDI-2026-2388640-s001.pdf]

CARE Checklist of information to include when writing a case report

CARE case report guidelines

(cc) BY-NC-ND

Topic	Item	Checklist item description	Reported on Line
Title	1	The diagnosis or intervention of primary focus followed by the words "case report"	
Key Words	2	2 to 5 key words that identify diagnoses or interventions in this case report, including "case report"	
Abstract (no references)	3a	Introduction: What is unique about this case and what does it add to the scientific literature?	
	3b	Main symptoms and/or important clinical findings	
	3c	The main diagnoses, therapeutic interventions, and outcomes	
	3d	Conclusion—What is the main "take-away" lesson(s) from this case?	
Introduction	4	One or two paragraphs summarizing why this case is unique (**may include references**)	
Patient Information	5a	De-identified patient specific information.	
	5b	Primary concerns and symptoms of the patient.	
	5c	Medical, family, and psycho-social history including relevant genetic information	
	5d	Relevant past interventions with outcomes	
Clinical Findings	6	Describe significant physical examination (PE) and important clinical findings.	
Timeline	7	Historical and current information from this episode of care organized as a timeline	
Diagnostic Assessment	8a	Diagnostic testing (such as PE, laboratory testing, imaging, surveys).	
	8b	Diagnostic challenges (such as access to testing, financial, or cultural)	
	8c	Diagnosis (including other diagnoses considered)	
	8d	Prognosis (such as staging in oncology) where applicable	
Therapeutic Intervention	9a	Types of therapeutic intervention (such as pharmacologic, surgical, preventive, self-care)	
	9b	Administration of therapeutic intervention (such as dosage, strength, duration)	
	9c	Changes in therapeutic intervention (with rationale)	
Follow-up and Outcomes	10a	Clinician and patient-assessed outcomes (if available)	
	10b	Important follow-up diagnostic and other test results	
	10c	Intervention adherence and tolerability (How was this assessed?)	
	10d	Adverse and unanticipated events	
Discussion	11a	A scientific discussion of the strengths AND limitations associated with this case report	
	11b	Discussion of the relevant medical literature **with references**.	
	11c	The scientific rationale for any conclusions (including assessment of possible causes)	
	11d	The primary "take-away" lessons of this case report (without references) in a one paragraph conclusion	
Patient Perspective	12	The patient should share their perspective in one to two paragraphs on the treatment(s) they received	
Informed Consent	13	Did the patient give informed consent? Please provide if requested	Yes ☐ No ☐

CARE Checklist - Completed Section

- Title - Item 1: Page 1, Title: "Emerging Threat of Acinetobacter Radioresistens Infection in Immunocompromised Patients"
- Key Words - Item 2: Page 1, Keywords: "Acinetobacter *radioresistens*, Bacteremia, VERIGENE, MALDI-TOF, Antimicrobial Resistance"

Abstract

- 3a: Highlights the rarity of A. radioresistens bacteremia and its diagnostic challenges.
- 3b: "acute hypoxic respiratory failure and septic shock."
- 3c: Identification methods, treatment with ampicillin-sulbactam, initial response, and transition to comfort care.
- 3d: Clinical significance in immunocompromised hosts and the utility of rapid diagnostics.
- Introduction - Item 4: Discusses A. radioresistens as an emerging opportunistic pathogen and the importance of this case.

Patient Information

- 5a: "77-year-old male"
- 5b: "shortness of breath"
- 5c: "adenocarcinoma of the lung and liver metastasis... COPD, dementia, and history of alcohol abuse."
- 5d: "recent cancer diagnosis, no chemotherapy or radiation yet."
- Clinical Findings - Item 6: "afebrile, tachypneic (31 breaths/min), hypoxic (85%), hypotensive (77/61 mm Hg), crackles in upper right lung."
- Timeline - Item 7: Case Presentation, Paragraph 4: "A detailed timeline... is provided in Table 1."

Diagnostic Assessment

- 8a: Labs, imaging, microbiological testing (Gram stain, VERIGENE, MALDI-TOF, VITEK 2).
- 8b: Identification challenges with rare pathogens, confirmatory testing.
- 8c: A. radioresistens bacteremia; considered pneumonia due to Klebsiella pneumoniae.
- 8d: Poor prognosis due to metastatic disease, comorbidities, mortality up to 70%.

Therapeutic Intervention

- 9a: Antibiotics (vancomycin, cefepime, ampicillin-sulbactam, metronidazole), mechanical ventilation, vasopressors.
- 9b: Dosages: vancomycin 1000 mg IVPB, cefepime 1000 mg IVPB, ampicillin-sulbactam 3 g q8h (8 days), metronidazole 500 mg q8h (2 days).
- 9c: Switched antibiotics after susceptibility results (pan-susceptible).

Follow-up and Outcomes

- 10a: Initial improvement, weaned off supports, later hypoxemic failure, hospice care, expired.
- 10b: Recurrence of hypoxemic respiratory failure.
- 10c: Implied adherence via initial improvement.
- 10d: Sudden hypoxemic respiratory failure.

Discussion

- 11a: Strength: rapid antibiotic response; Limitation: one positive culture, concurrent K. pneumoniae.
- 11b: References cited (2, 3, 4, 5, 6, 15–18).
- 11c: Supported by culture results, severity, and response.
- 11d: Takeaway: significance of pathogen and diagnostics importance.
- Patient Perspective - Item 12: Not applicable (dementia, expired; family elected comfort care).
- Informed Consent - Item 13: No.
